# Unpredictable Drug Access and its Relevance for Substance Use Disorders: A Critical Review

**DOI:** 10.1007/s40614-025-00449-1

**Published:** 2025-06-02

**Authors:** William S. Doyle, Kevin B. Freeman, Sally L. Huskinson

**Affiliations:** 1 Program in Neuroscience, University of Mississippi Medical Center, Jackson, MS USA; 2 Department of Psychiatry and Human Behavior, University of Mississippi Medical Center, 2500 N. State Street, Jackson, MS 39216 USA; 3 Center for Innovation and Discovery in Addictions, University of Mississippi Medical Center, Jackson, MS USA

**Keywords:** Behavioral pharmacology, Substance use disorders, Unpredictable drug access, Discounting framework, Laboratory models

## Abstract

Many factors contribute to drug use and the development of substance use disorders (SUDs). We and others have argued that individuals who misuse illicit drugs face circumstances in which their access to drugs is variable or unpredictable, particularly for those who are experiencing poverty. Herein, we make the case that such unpredictable drug access increases drug intake and choice, particularly when the time and effort required to obtain drugs is relatively large. If translated to real-world scenarios, unpredictable access could lead to increased severity of drug misuse, poorer treatment outcomes, persistence of drug seeking during periods of drug unavailability, drug taking despite negative consequences, and increased relapse rates, though additional research is surely needed. We describe how variable drug access can be evaluated in the laboratory, review prior research that has compared variable vs. fixed drug access, provide a summary of preclinical findings based on the literature reviewed, and end with implications for policy and treatment for individuals with SUDs.

Several environmental and genetic factors contribute to drug use, misuse, and the development of substance use disorders (SUDs). In the current review, we focus on one environmental factor that may contribute to drug-use patterns and the development of SUDs: unpredictable access to illicit drugs. We and others have argued that individuals who misuse illicit drugs face circumstances in which the time and effort required to obtain drugs, drug quality, and durations of use and alternating periods of abstinence are variable or unpredictable (see Doyle & Huskinson, 2023; Huskinson, 2020 for prior reviews). Herein, we make the case that such unpredictable drug availability increases drug intake and choice, particularly when the time and effort required to obtain drugs is relatively large. If translated to real-world scenarios, unpredictable availability could lead to increased severity of drug misuse, poorer treatment outcomes, persistence of drug seeking during periods of drug unavailability, drug taking despite negative consequences, and increased relapse rates, though additional research is needed in this area. We describe various ways in which variable drug delivery can be evaluated in the laboratory, review prior research that has compared variable vs. fixed drug access in human participants and nonhuman subjects, provide a summary and conclusion based on the literature reviewed, and end with implications for policy and treatment for individuals with SUDs.

For those who use illicit drugs, the consistency of drug availability likely exists on a continuum, varying in terms of effort required, quantity available, quality of the drug product, or delays in access. At one end of the continuum, for individuals with a SUD who live in resource-rich environments, who have relatively high incomes with stable employment and housing, likely have access to illicit drugs under more predictable time and effort requirements, for predictable amounts of drug, and for predictable periods of time. At the other end of the spectrum are individuals with a SUD who live in resource-scarce environments, with lower incomes and a lack of stable employment and housing. Individuals under these circumstances likely have access to illicit drugs under more unpredictable time and effort requirements, for unpredictable amounts of drug, and for unpredictable periods of time. Furthermore, those experiencing resource scarcity likely experience large time and effort requirements for the purchase of small amounts of drug at a time because these individuals do not have the funds to purchase large amounts compared with those in a resource-rich environment. This may result in individuals with SUDs who live in poverty spending most of their time trying to obtain sufficient funds to purchase drugs, and the cycle of drug procurement may repeat often because small quantities are purchased at a time. The amount of drug purchased at a time subsequently determines how long drug use persists before entering a period of drug unavailability, at which time the individual must acquire additional funds to purchase more drugs.

Similarly, the predictability of competing nondrug alternatives in an individual’s environment depends on their available resources. Those experiencing a scarcity of resources have relatively unpredictable access to nondrug commodities like reliable meals, transportation, or income. These individuals with a SUD are faced with difficult scenarios in which their limited funds must be allocated among available drug and nondrug commodities, and drug seeking may garner a disproportionate amount of their behavior. Indeed, poverty is associated with increased prevalence and severity of substance misuse (e.g., Armstrong, 2007; Center for Behavioral Health Statistics & Quality 2018; Silverman et al., 2020; Williams & Latkin, 2007). Furthermore, those experiencing homelessness use substances at a higher-than-average rate (e.g., Fazel et al., 2008), and, among patients who visit emergency departments for their healthcare needs, those experiencing current or past homelessness show higher rates of substance use and greater severity of use compared with those who have not experienced homelessness (Doran et al., 2018). It is possible that, among several driving factors, the unpredictability of illicit substances and nondrug commodities in impoverished conditions exacerbates substance use and associated harms.

Unfortunately, there are very few investigations that identify the predictability or unpredictability that individuals actually experience in the acquisition and use of their illicit substances. Ward and colleagues (1997) reported that most participants who use cocaine described binges in use that were separated by breaks because of a need to “hustle” to get enough money to buy more cocaine. In a survey of self-reported changes in drug and alcohol use after becoming homeless, individuals self-reported their means of acquiring drugs (O’Toole et al., 2004). These individuals reported diverting funds from daily sustenance or using rent or food money, selling or trading food stamps, selling one’s belongings, working for a dealer in exchange for drugs, and stealing. About half of the participants who use cocaine and heroin reported panhandling and begging for money. These individuals also reported diverting child support money or exchanging sex for drugs, though this was the least common means of acquiring drugs among these participants (O’Toole et al., 2004). These self-reports do not explicitly note that their means of acquiring substances were unpredictable. However, acquiring substances in the manner that was reported most likely is unpredictable in that periods of use depend on the amount of money acquired, and subsequently, the amount of drug purchased at a time. Similarly, breaks in use likely depend on the time taken to obtain more money to purchase additional drugs. Altogether, such unpredictable conditions would leave less time for an individual to allocate their behavior toward the pursuit and consumption of nondrug commodities in the environment and ultimately may lead to worsened drug-use patterns and SUD outcomes. Indeed, participants reported giving up nondrug commodities in exchange for substances (O’Toole et al., 2004).

Illicit drugs also are unpredictable in terms of quality because cutting agents or adulterants are mixed into these supplies (e.g., Broséus et al., 2016; Cole et al., 2011; Fiorentin et al., 2019). Individuals report getting “blanks” or poor-quality drugs and estimate a wide range of purity of their supply (2–100%; McNeil et al., 2022; Roddy & Greenwald, 2009; Roddy et al., 2011; Szalavitz, 2016). These anecdotes are supported by seized drugs that vary widely in terms of purity (e.g., Fiorentin et al., 2019). In a recent Drug Checking Report (Krotulski et al., 2022, Q3) from Philadelphia, the purity of seized cocaine averaged 38% (range: 0.2–81.8%). Furthermore, in recent years, illicit opioids and stimulants have been adulterated with fentanyl (e.g., Ciccarone, 2017; Ciccarone et al., 2017; DEA, 2021; Zibbell, 2019), resulting in a different and more dangerous type of adulterated and unpredictable supply as fentanyl is a major contributor to the overdose crisis and rise in deaths in the U.S. (Ciccarone, 2019; CDC).

Research in our preclinical laboratory has focused on the impact that unpredictable drug access has on drug self-administration and drug choice, as well as the implications of these laboratory outcomes for individuals with SUDs. In the next sections, we describe several ways in which unpredictable drug access can be modeled and evaluated in the laboratory and review the studies that have evaluated unpredictable drug access in human and nonhuman subjects. It is important to note that there is a large literature on the behavioral and neurobiological consequences of unpredictable delivery of nondrug rewards and reinforcers; and while outcomes with nondrug delivery generally are similar to that reported for drugs, the focus of the current review is on drug rewards and reinforcers.

## Variable Response Requirements

One way to model variability in the laboratory is to manipulate the schedule of reinforcement, arguably mimicking the time and effort an individual must invest in obtaining a drug. The most common way that this has been done has been to use a variable-ratio (VR) or random-ratio (RR) schedule of reinforcement. These schedules result in an unpredictable number of responses required for each reinforcer delivery, though the manner in which they are programmed is slightly different. Under a VR schedule, a variable number of responses is required for each reinforcer, and the average number of responses per delivery is equal to a predetermined value. Under an RR schedule, each response has a predetermined probability of delivering a reinforcer, and typically, over a large number of trials, the average number of responses per reinforcer is equal to a predetermined value. For example, under an RR 20 schedule, each response has a 5% chance of resulting in a reinforcer. VR and RR schedules can be compared with fixed-ratio (FR) schedules that always require the same number of responses per reinforcer. We argue that VR and RR schedules model unpredictable drug access in that drug-seeking sometimes results in more immediate access to drugs but sometimes requires a large amount of time and effort, resulting in more delayed access to drugs.

A handful of experiments have evaluated how unpredictable response requirements affect drug taking. Using a behavior-economic procedure in rhesus monkeys, Lagorio and Winger (2014) gave rhesus monkeys access to cocaine, remifentanil (an ultra-short acting mu-opioid agonist), or ketamine under FR or RR schedules of reinforcement. In their experiment, RR schedules resulted in higher rates of responding, higher intakes, and greater demand for all three drugs compared with FR schedules. This effect was most pronounced with larger response requirements and lower doses, indicating that unpredictability in high-cost and low-magnitude reinforcement conditions, like those most likely to occur for individuals living in poverty, exert the greatest effect on drug-taking behavior. Furthermore, increased drug self-administration under RR vs. FR schedules were more robust for cocaine and remifentanil compared with ketamine, suggesting that unpredictable access to drugs may exert a stronger effect on drug taking with more effective drug reinforcers (i.e., cocaine and remifentanil) compared with less effective drug reinforcers (i.e., ketamine).

**Fig. 1:**
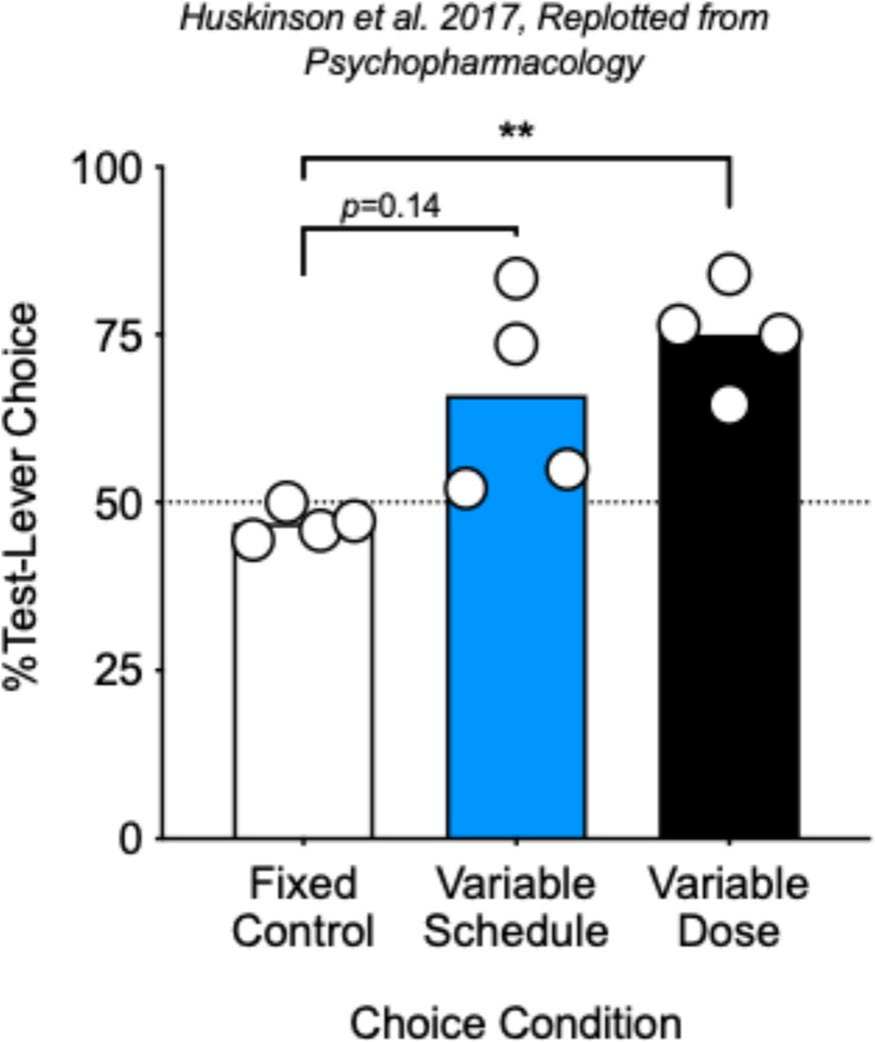
Select data from Fig. 1 in Huskinson et al., (2017) are replotted. Mean percent test-lever choice as a function of the choice condition, either Fixed Control (open bar) when both cocaine doses were 0.1 mg/kg/injection and both schedules were FR 30; Variable Schedule (blue/shaded bar) when both cocaine doses were 0.1 mg/kg/injection and one schedule was an FR 30 and the other a VR 30; and Variable Dose (filled bar), when one cocaine dose was fixed at 0.1 mg/kg/injection and the other was variable at 0.02, 0.1, or 0.18 mg/kg/injection and both schedules were an FR 30. Circles represent individual male subjects, and ** are significant differences compared to fixed control at *p* < 0.01. See text for additional details

In our laboratory, we largely have focused on choice behavior, or the allocation of behavior among available outcomes in the environment; and therefore, much of our work in this area has been to evaluate choice between drug and nondrug reinforcers under FR and VR schedules. Figure 1 shows select results from our first experiment in which male rhesus monkeys (*N* = 4) chose between the same dose of cocaine (0.1 mg/kg/injection) available following completion of the response requirement on either one of two levers. In different conditions, cocaine was available under equal FR 30 schedules on both response options (open bar, Fig. 1) or under a VR 30 schedule on one option and an FR 30 schedule on the other option (blue/shaded bar, Fig. 1; Huskinson et al., 2017). As expected, average choice was indifferent when the same cocaine dose was available under equal FR 30 requirements. However, when one option was available under a VR 30 and the other under an FR 30, two of four subjects reliably chose cocaine associated with the VR option more than that associated with the FR option.

**Fig. 2:**
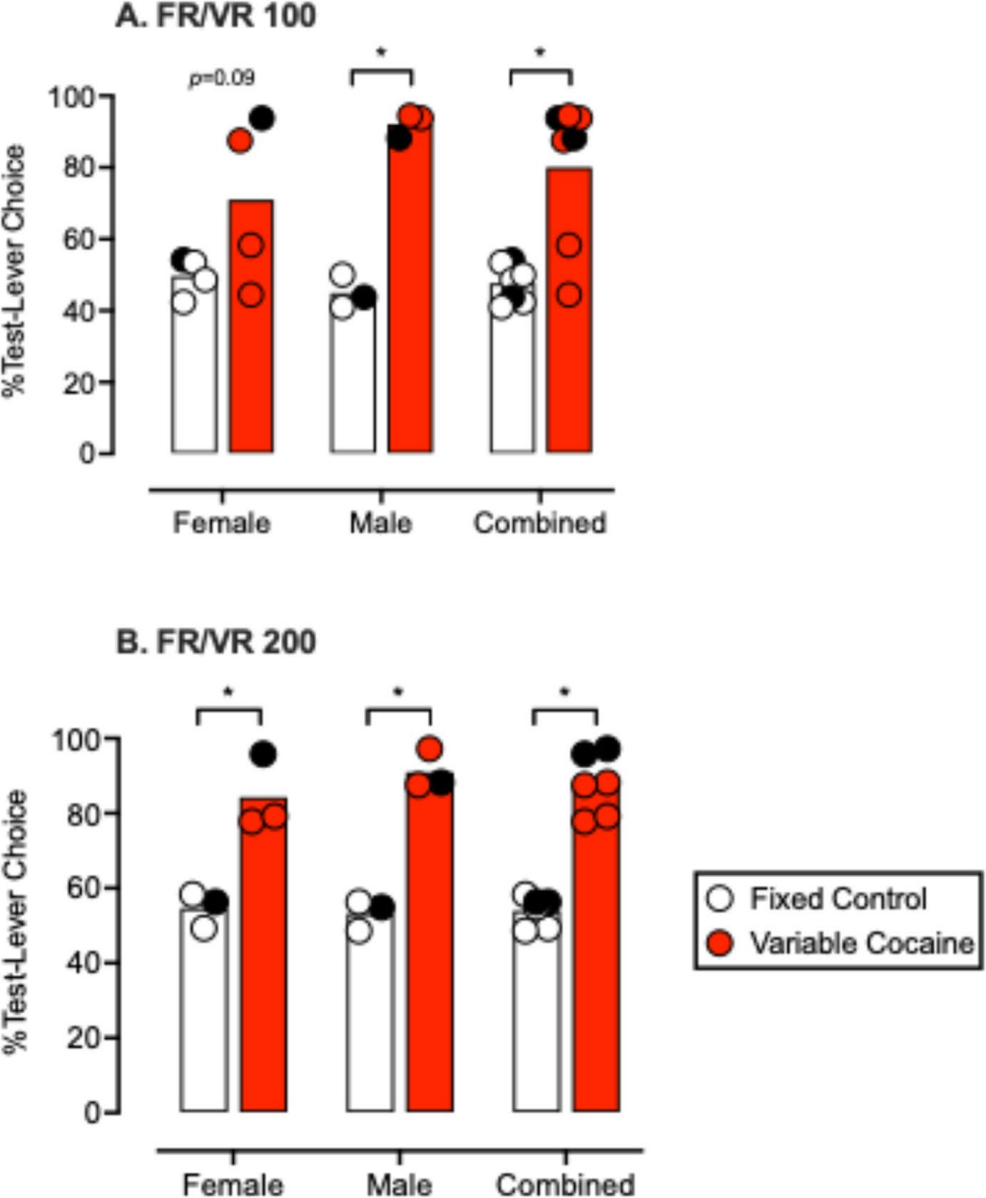
Data and figure from Zamarripa et al., (2023, original Fig. 2). Reprinted with permission. Mean percent test-lever choice for females, males, and both groups combined during cocaine (0.018 or 0.03 mg/kg/injection) vs cocaine (0.018 or 0.03 mg/kg/injection) choice conditions. Data points represent the means for individual subjects from the final 3 stable sessions for each lever pairing and its reversal, and bars represent the group means. The solid data points represent the one female (71–2006) and one male (69–2012) that experienced these conditions with a smaller dose of cocaine (0.018 mg/kg/injection). Open bars and circles represent the Fixed-Control conditions, and red or shaded bars and circles represent the Variable-Cocaine conditions when responding was maintained under an FR/VR 100 (Panel A) or an FR/VR 200 (Panel B). Asterisks indicate significant differences at *p* < 0.05

Figure 2 shows results from a later experiment (original Fig. 2 from Zamarripa et al., 2023), when the response requirement was larger (i.e., 100 or 200), and data are shown separated by sex and for all subjects combined. As in the earlier experiment, choice was indifferent when equal cocaine doses were available under equal FR 100 or FR 200 requirements (open bars, Fig. 2). However, when one option was available under a VR 100 and the other under an FR 100 (Fig. 2, panel A), two of four females and all three males chose cocaine associated with the VR option more than that associated with the FR option. Finally, when cocaine was available under FR and VR 200 schedules (Fig. 2, panel B). All three female and all three male subjects chose cocaine associated with the VR 200 schedule over that associated with the FR schedule.

It is important to note that in our later experiment, we evaluated cocaine vs. cocaine choice under slightly different parameters (Zamarripa et al., 2023). First, the number of available choices per session was raised to 18 or 24 vs. 12 choices per session in our prior report (Huskinson et al., 2017). Second, the available cocaine doses were lower (0.018 or 0.03 mg/kg/injection), and the timeout between choice trials was shorter (i.e., 5 min vs. 20 min). Finally, we included both male and female subjects. Despite these differences in experimental conditions, and like that observed with behavior-economic demand (Lagorio & Winger, 2014), choice of a VR-associated option was more robust with larger average response requirements (i.e., high-cost conditions) vs. smaller requirements, which are conditions most likely to be experienced by individuals living in poverty.

In the same experiment, we evaluated choice between cocaine (0.01–0.1 mg/kg/injection) and food (4 pellets/delivery) under FR and VR 100 and 200 schedules (Zamarripa et al., 2023). In these experiments, cocaine choice increased when cocaine was available under a VR 100 or 200 schedule and food was available under an FR 100 or 200 schedule compared to control conditions when both options were available under FR 100 or 200 schedules. We also evaluated choice when cocaine was available under an FR 100 or 200 schedule and food was available under a VR 100 or 200 schedule. Under these conditions, food choice increased (and cocaine choice decreased) when the average schedule value was 200 but not when the schedule values were 100. These outcomes were interesting because they suggested that drug choice may be more sensitive to variable response requirements (i.e., at lower overall cost requirements) than nondrug choice. Another caveat to these results is that the most robust differences between FR and VR schedules tended to occur in the middle of the cocaine dose–response curve, less so with smaller doses, and not at all with larger doses.

In another recent experiment (Doyle et al., 2023), we were interested in extending our results with cocaine to fentanyl and wanted to determine whether our outcomes in drug vs. nondrug choice could be evaluated in a more rapid manner using a within-session choice procedure in which complete dose–response curves can be determined within a session (e.g., Negus, 2003; Paronis et al., 2002). Using this procedure, the amount of food available on one lever remained fixed (2 pellets/delivery) across five choice components, and the cocaine or fentanyl dose was zero in component one and increased across choice components two to five. In this experiment, the schedule of cocaine or fentanyl reinforcement was either an FR or VR 100 and the schedule of food reinforcement was an FR 25, 50, or 100 in different conditions. Under these conditions, cocaine choice increased under the VR schedule compared with the FR schedule, but only when food was available under an FR 50 or 100 schedule. When food was available under an FR 25 schedule, cocaine choice was unaffected by schedule type, suggesting that effects of unpredictable access to cocaine can be ameliorated by making nondrug alternatives less costly. For fentanyl, drug choice was enhanced under the VR schedule, independent of the size of the schedule of reinforcement for food. It should be noted, however, that outcomes with fentanyl were less robust across the board compared with cocaine, and while outcomes were statistically significant, effect sizes were much smaller with fentanyl vs. cocaine. Finally, as was the case in our prior report (Zamarripa et al., 2023), differences in cocaine or fentanyl choice tended to occur in the middle of the dose–response curve rather than at the extremes (i.e., with small or large doses).

In a different evaluation of effects of exposure to VR schedules on drug self-administration, Mascia and colleagues (2019, 2020) demonstrated that rats with a history of responding under VR schedules of saccharin reinforcement subsequently had higher rates of amphetamine self-administration under a progressive-ratio (PR) schedule of reinforcement than rats with a history of responding under FR schedules of saccharin reinforcement. This outcome suggests that a history of unpredictable access to a nondrug reinforcer can increase the reinforcing efficacy of drug reinforcers. These subjects exposed to VR schedules of nondrug reinforcement also showed enhanced sensitivity to amphetamine-induced locomotor activity, a finding that others have shown (e.g., Singer et al., 2012; Zack et al., 2014; Zeeb et al., 2017) compared with subjects exposed to FR schedules of nondrug reinforcement. Furthermore, dopamine (DA) overflow in the nucleus accumbens was greater in rats responding under the VR schedule during saccharin reinforcement compared with those responding under the FR schedule, particularly at larger response requirements compared with smaller ones. These authors demonstrated that several other neurobiological outcomes differed between animals exposed to VR vs. FR schedules of nondrug reinforcement, including for example, changes in aspartic acid and glutamate expression in the nucleus accumbens (Mascia et al., 2020).

## Variable Magnitude

As indicated above, another unpredictable aspect of the illicit drug supply is variability in drug quality. This can be modeled in the laboratory by manipulating the dose of drug delivered per reinforcer (i.e., fixed dose vs. variable dose). In many traditional drug self-administration studies, completion of each response requirement results in delivery of a predictable and fixed dose. However, in a variable-dose arrangement, completion of each response requirement results in the delivery of a range of drug doses, including those that are smaller or larger than the predetermined average dose. There are a handful of experiments that have been conducted in which fixed vs. variable dosing were directly compared in drug self-administration. Wise and colleagues (1995) allowed rats to self-administer either a fixed (2.0 mg/kg/injection) or variable (0.5, 1.0, or 2.0 mg/kg/injection) dose of cocaine, and nucleus accumbens DA levels were monitored via microdialysis. While DA levels were elevated significantly during self-administration under both conditions, variable-dose injections were followed by greater increases in DA than fixed-dose injections.

The only other research, to our knowledge, in which fixed vs. variable dosing were directly compared in nonhuman subjects is from our previously mentioned study (Huskinson et al., 2017). Figure 1 also shows the condition in which choice was between a fixed dose of cocaine available under an FR 30 schedule and variable doses of cocaine, also available under an FR 30 schedule. When both doses were equal, on average, all subjects chose the variable dose over the fixed dose (Fig. 1, black/filled bar). Furthermore, when the variable dose was lower, on average, than the fixed dose, one subject continued to choose the variable dose over the fixed dose (data not shown).

Additionally, there are two studies (Bickel et al., 2004; Kirshenbaum et al., 2006) in which opioid-dependent human participants chose between hypothetical fixed and variable sources of heroin. Bickel and colleagues (2004) determined that, in a simulated state of withdrawal, but not in a simulated state of satiation, both variable amounts of heroin and variable potencies per bag of heroin were chosen over their fixed counterparts, a phenomenon that was most robust when the average amount of bags or potency was the greatest (i.e., high-magnitude conditions). The second study, conducted by Kirshenbaum and colleagues (2006), reported similar results with fixed vs. variable amounts of an opioid. As reported by Bickel and colleagues (2004), a simulated state of opioid withdrawal enhanced choice of variable over fixed hypothetical opioid amounts, and this difference was more robust in individuals who use opioids intravenously compared with those who use intranasally. However, Kirshenbaum and colleagues did not replicate the finding that choice of the variable option increased as a function of the magnitude of hypothetical opioids. In fact, the opposite relation was true, where choice of the variable option decreased as magnitude increased. Interestingly, a simulated state of opioid withdrawal also resulted in increased choice of a variable monetary outcome over a fixed monetary outcome for both intravenous and intranasal users (Kirshenbaum et al., 2006). Altogether, results with variable magnitudes suggest that unpredictability in the quality of a drug supply can enhance drug choice over more predictable supplies.

## Variable Delay to Drug Access

Illicit-drug users also may experience variable delays to reinforcer delivery. To our knowledge, there are no prior reports in which variable vs. fixed delays to drug delivery were evaluated in nonhuman subjects. However, there are examinations of variable vs. fixed delays to hypothetical drugs in human participants. Two of these studies were described above in our discussion of variable magnitudes (Bickel et al., 2004; Kirshenbaum et al., 2006). In addition to the previously described variable amounts and potencies, variable delays were consistently chosen over fixed delays, particularly in simulated states of opioid withdrawal compared with simulated states of opioid satiation. In addition, choice of the variable option occurred more robustly with longer delays compared with shorter delays, such that even in a simulated state of opioid satiation, longer variable delays were chosen over shorter fixed delays (Bickel et al., 2004). Like with fixed vs. variable magnitudes, outcomes were more robust in intravenous users compared with intranasal users (Kirshenbaum et al., 2006).

In addition, Schlagintweit et al. (2015) evaluated variable delay to nicotine reinforcement by providing anticipated vs. unanticipated smoking opportunities. In the “anticipated” group, participants were informed that they would receive an opportunity to smoke during the study, whereas participants in the “unanticipated” group were told they would not be able to smoke during the study but were later provided the opportunity. While overall intake did not differ between groups, the latency to smoke was significantly shorter for the “unanticipated” group compared with the “anticipated” group, and the authors concluded that smoking may be more appealing when smoking opportunities are unanticipated.

## Variable Stimuli

During the course of drug use, certain environmental stimuli (e.g., location, time, people) are reliably paired with drug taking and, through conditioning, become directly associated with the effects of the drug itself. Because these environmental stimuli are regularly paired with drug taking, they may elicit craving through a compensatory conditioned response. To our knowledge, there is one investigation in which predictable or unpredictable pairing of stimuli to drug or saline delivery was evaluated. D’Souza and Duvauchelle (2008) determined the influence of unpredictable stimulus presentations on DA overflow in the nucleus accumbens during a cocaine or saline self-administration event. For one group of rats, predictable stimuli were present during cocaine vs. saline availability in drug self-administration training, and for the other group, unpredictable stimuli were present during periods of cocaine vs. saline availability during training. During training, locomotor activity was greatest for rats in the unpredictable group compared with the predictable group during both cocaine and saline self-administration sessions. Furthermore, nucleus accumbens DA overflow increased to a greater degree following cocaine delivery in the group that received unpredictable stimulus pairings compared with those in the group that received predictable stimulus pairings. These results, like prior outcomes with variable response requirements or variable doses, suggest that excessive DA overflow in reward pathways is likely an important determinant of enhanced choice of drugs available under unpredictable arrangements.

## Variable Access and No-Access Durations

In recent years, there has been an increasing interest in intermittent access drug self-administration procedures (e.g., Kawa et al., 2016; Zimmer et al., 2012). In these studies, fixed periods of access to a drug reinforcer predictably alternates with fixed no-access periods. When compared with continuous-access conditions, nonhuman subjects generally self-administer more drug under intermittent compared with continuous conditions despite having less time to respond for the drug reinforcer during intermittent access. We are aware of only one study in which a fixed, intermittent-access procedure was compared to a variable, intermittent-access procedure in which alternating periods of access and no-access were of variable durations (Robinson et al., 2023). This experiment, however, evaluated nondrug reinforcers and combined variable access with a variable schedule, variable magnitude, and probabilistic delivery. As expected, the variable conditions resulted in higher levels of responding, and in subsequent conditions, resulted in greater progressive-ratio and extinction responding as well as cue-induced reinstatement maintained by a nondrug reinforcer. It will be important for future studies to evaluate whether a variable, intermittent-access procedure, in which only the duration of access and no-access periods are variable, would result in similar outcomes with a drug reinforcer compared with that reported with nondrug reinforcers.

## Summary of Preclinical Findings

Herein, we reviewed several studies in which real and hypothetical drug rewards or reinforcers were made predictably or unpredictably available to human participants or nonhuman subjects. The manner in which drugs were made predictable or unpredictable included variable response requirements, magnitudes of delivery, delays to delivery, and variable stimulus conditions. While no prior research has evaluated fixed vs. variable intermittent-access procedures with drug reinforcers, this procedure models unpredictable drug access and future research should be done with this model with drug reinforcers. The studies reviewed indicate that unpredictable access to drugs can result in detrimental effects on drug taking and the neurobiological outcomes associated with drug taking like excessive DA release. Importantly, however, the research reviewed also demonstrated that such detrimental effects are dependent on several environmental conditions.

With variable schedules or delays to reinforcement, the most robust differences were observed when the response requirement or delay to reinforcement was large or long, respectively, compared to conditions when the response requirement or delay was small or short (e.g., Bickel et al., 2004; Huskinson et al., 2017; Kirshenbaum et al., 2006; Lagorio & Winger, 2014; Mascia et al., 2019, 2020; Zamarripa et al., 2023). In addition, outcomes tended to be dose-dependent in that small to moderate doses resulted in the greatest differences between FR and VR or RR schedules whereas little to no differences were obtained with larger drug doses (Doyle et al., 2023; Huskinson et al., 2017; Lagorio & Winger, 2014; Zamarripa et al., 2023). Altogether, greater time and effort requirements and longer delays to the acquisition of drugs as well as obtaining small quantities of drug at a time are most likely to be the case for individuals living in poverty—individuals in most need of access to treatment and nondrug resources.

Individuals living in poverty also are more likely to have access to nondrug commodities under similarly unpredictable conditions. Interestingly, Mascia and colleagues (2019, 2020) demonstrated that a history of responding for nondrug reinforcers under a VR schedule enhances the reinforcing effectiveness of amphetamine compared to a history of responding for nondrug reinforcers under an FR schedule. If translated to humans, those experiencing unpredictable access to nondrug alternatives in their environment also may show an enhanced sensitivity to drug reinforcement. In further support of the need to make nondrug resources more cheaply available to individuals living in poverty, we demonstrated that making food available under a smaller response requirement ameliorated the enhanced choice of cocaine when available under a VR schedule compared with an FR schedule (Doyle et al., 2023).

Most of the work reviewed evaluated cocaine as the drug reinforcer. However, in a few experiments, an opioid was evaluated under FR vs. VR schedules (Doyle et al., 2023) or fixed vs. variable delays to reinforcement (Bickel et al., 2004; Kirshenbaum et al., 2006). It should be noted that in our preclinical experiment with rhesus monkeys, fentanyl choice when available under a VR schedule was statistically greater than fentanyl choice when available under an FR schedule, but outcomes were not as robust as those with cocaine. In contrast to stimulants like cocaine (Banks & Negus, 2010), physical dependence and its associated withdrawal are strong drivers of drug choice with opioids like heroin (e.g., Negus, 2006; Negus & Rice, 2009). When opioid-dependent human participants made choices between fixed and variable delays to hypothetical heroin, choice of the variable option was much more robust under longer variable delays compared with shorter ones, in simulated states of withdrawal compared with simulated opioid satiation, and in intravenous users compared with intranasal users (Bickel et al., 2004; Kirshenbaum et al., 2006). These outcomes suggest an enhanced sensitivity to variable outcomes in physically dependent individuals, and in those with riskier drug-use patterns. The rhesus monkeys in our prior experiment were not physically dependent (Doyle et al. 2023). Based on outcomes with humans, our results with fentanyl might have been more robust if subjects were physically dependent, and in particular, if choice had been assessed during a state of opioid withdrawal.

In an investigation of variable vs. fixed drug amounts, Wise and colleagues (1995) demonstrated greater DA overflow in the nucleus accumbens when variable cocaine doses were delivered compared with a fixed dose of cocaine. This result is similar to Mascia and colleagues’ (2019, 2020) outcomes with VR vs. FR schedules of saccharin delivery as well as fixed vs. variable nondrug reward amounts and in DA Prediction Error research (e.g., see Nasser et al., 2017; Schultz, 2016 for recent discussions of DA prediction error). These outcomes also are consistent with the one experiment in which unpredictable vs. predictable stimulus conditions resulted in excessive DA overflow in the nucleus accumbens following a self-administered cocaine injection (D’Souza & Duvauchelle, 2008). It is possible that the excessive DA overflow is the neural mechanism responsible for the outcomes reviewed in which drug-taking behavior was maintained under any of the fixed vs. variable conditions considered here.

Regarding variable magnitudes, similar results were obtained with human participants choosing between fixed and variable amounts of hypothetical heroin in which variable amounts and potencies of heroin were chosen over fixed ones, particularly during a simulated state of opioid withdrawal (Bickel et al., 2004; Kirshenbaum et al., 2006). Based on the research reviewed, however, it is unclear whether outcomes with fixed vs. variable doses are more robust with smaller or larger average doses. Indeed, outcomes with human participants are mixed, as one experiment (Bickel et al., 2004) demonstrated a positive relation between average dose or amount and choice of the variable option, whereas the other experiment (Kirshenbaum et al., 2006) did not replicate the same relation, and in fact, showed a negative relation between average dose or amount and choice of the variable option. Furthermore, a wide range of doses was not evaluated in rhesus monkeys (Huskinson et al., 2017). Future research is needed to determine the relation between drug dose and choice of fixed vs. variable options.

### Potential Behavioral

**Fig. 3:**
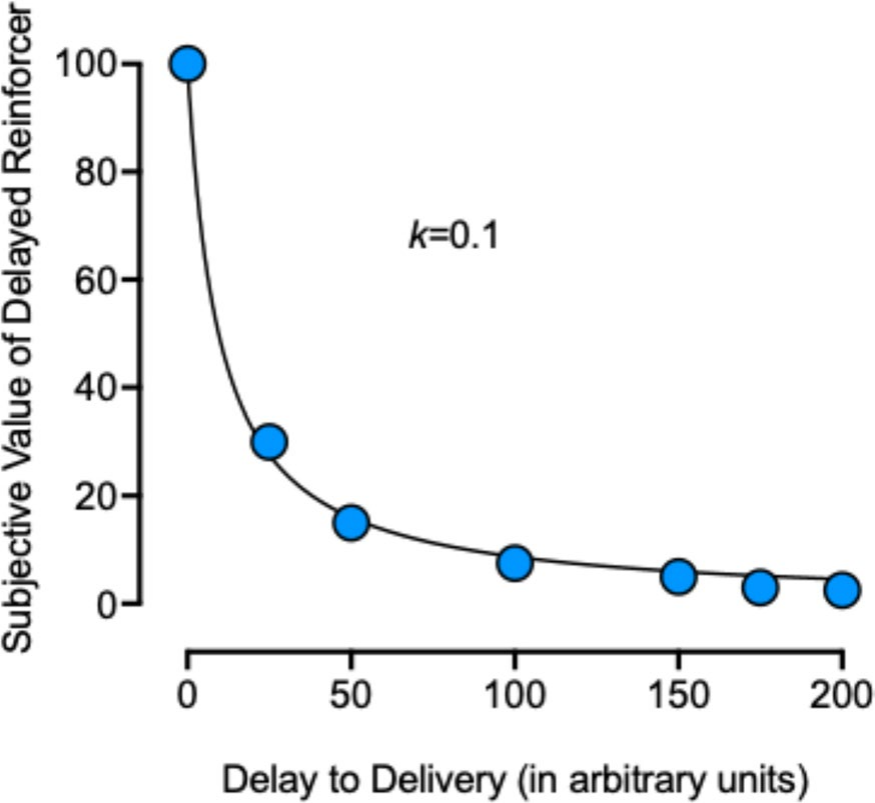
A hypothetical discounting function is shown by plotting hypothetical subjective values of a delayed reinforcer (y-axis) across hypothetical delays to reinforcer delivery (x-axis). Data points were fit with the Mazur’s (1987) hyperbolic equation V = A/(1 + *k*D), where V is the present value of the delayed reinforcer, A is the magnitude of the delayed reinforcer, *k* is a free parameter that reflects discounting rate, and D is delay to reinforcer delivery. Data points on the y-axis are 100, 30, 15, 7.5, 5, 3, 2.5 at x-axis delays of 0, 25, 50, 100, 150, 175, and 200, respectively. The resulting *k* value of the curve is 0.1, with *R*^*2*^ = 0.99

What is missing for all the dimensions of unpredictability evaluated are behavioral mechanisms that may be responsible for the outcomes reviewed, and future studies that target potential behavioral mechanisms are needed. We have described potential mechanisms in prior reviews (see Huskinson, 2020) and will therefore limit the current review to a brief description of one behavioral mechanism that we find particularly compelling. It is well established that the subjective value of a reinforcer decreases relatively rapidly at shorter delays and less rapidly with longer delays, taking a hyperbolic shape (e.g., Mazur, 1987). As a result, the combined value of a reinforcer available under a variable range of response requirements or delays to reinforcement, is subjectively greater than the value of the fixed but always delayed reinforcer under a fixed schedule or delay to reinforcement. Figure 2 shows a hypothetical discounting function to illustrate the point made previously by Madden and colleagues (2007, 2011; also see Huskinson, 2020). The value of a reinforcer, ranging from 0 to 100, is plotted as a function of delay to its delivery in arbitrary units. At a delay of 25 units, the subjective value when delivery is always fixed is 30. Conversely, using an equal on average, two-value variable schedule or variable delay at 0 and 50 units, the value at delay 0 is 100 and at delay 50 is 15. The sum of values at delay 0 and 50, multiplied by their probability of occurrence (*p* = 0.5), equals a combined subjective value of 57.5, a value that is greater than the subjective value of 30 at a fixed delay of 25 units. Thus, greater weight is applied to the sometimes-immediate delivery that can occur under variable schedules or delays to reinforcement, and proportionately less value is lost with the sometimes-delayed delivery that also occurs under variable schedules and delays (Fig. 3).

A discounting framework also can account for the finding that drug choice under variable vs. fixed schedules or delays to reinforcement become more extreme with larger schedules or delays. In the prior example, an arbitrary delay of 25 units was selected. In this example, the subjective value of the variable option was 57.5 and of the fixed option was 30. The difference in subjective values between these two options is therefore 27.5. Using a larger delay of 100 units, the subjective value when reinforcer delivery is fixed is 7.5, and the subjective values of the equal variable delays of 0 and 200 units are 100 and 2.5, respectively, for a combined subjective value of 51.25. Now, the difference between subjective values of the variable delay (51.25) and the fixed delay (7.5) is 43.75 and is greater than the difference obtained above of 27.5 with a delay of 25 units. In other words, the larger the schedule or delay to reinforcer delivery, the greater the difference in subjective values between variable vs. fixed reinforcer delivery. Importantly, the greater subjective value of variable schedules or delays to reinforcement compared with fixed schedules and delays also occurs when several values constitute the variable option (see Madden et al., 2007); two values were used in this example for simplicity.

Other common outcomes with fixed vs. variable schedules and delays with nondrug reinforcers also can be accounted for within a discounting framework, including the finding that choice of a variable option is greatest when the smallest possible response requirement is 1 or delay is 0, and the effect diminishes as the smallest possible value approaches that of the FR. Whether a discounting framework can account for outcomes with variable- vs. fixed-quality drug reinforcers or fixed vs. variable durations of access is unclear and will require a systematic exploration of a range of response requirements and reinforcer magnitudes or durations of access under fixed vs. variable conditions.

While no prior research has evaluated variable vs. fixed access and no-access periods in the context of drug self-administration, this pattern of behavior seems characteristic of the drug-seeking and -taking process for individuals who must “hustle” to earn sufficient funds to purchase drugs. It will be important for future research to determine whether variable and intermittent access to drug reinforcers increases overall drug intake and reinforcing efficacy of drugs in a manner similar to what has been reported with a nondrug reinforcer (Robinson et al., 2023).

Another key feature that is missing from all dimensions of variability reviewed is whether these experiences make abstinence more difficult to achieve or increase relapse-related behavior following a period of abstinence. No prior research has been conducted to determine whether unpredictable access to drug reinforcers exacerbates resistance to extinction or increases subsequent relapse-like behaviors (e.g., reinstatement, resurgence, renewal) compared with more predictable access. However, within the behavioral momentum field, there is a large body of work that has evaluated different schedules and magnitudes of reinforcement and their impact on extinction and relapse-like behaviors (see Nevin & Shahan, 2011 for a review of behavioral momentum) that could inform future investigations on fixed vs. variable access to drugs. Some of this work currently is underway in our laboratory, and these types of experiments will have important implications for abstinence and relapse in individuals whose access to illicit drugs is unpredictable.

### Implications for Policy and Treatment

We have reviewed policy and treatment implications of this type of research elsewhere (see Doyle & Huskinson, 2023 for a recent review), and therefore, only briefly review this area here. If outcomes reviewed above translate to real-world scenarios in which individuals use drugs in an illicit market, unpredictable access to drugs, particularly in resource-scarce environments like that experienced for individuals living in poverty, could lead to increased severity of drug misuse, poorer use patterns and treatment outcomes, persistence of drug seeking or taking during drug unavailability and despite negative consequences, and increased relapse rates. We have focused largely on how unpredictable access to drugs may worsen drug-related outcomes, but we have not focused on what this means for behavior maintained by predictable access to drugs.

Importantly, the research reviewed, if translated to real-world scenarios, also implies that predictable access to drugs makes drug-related behavior better. For example, cocaine choice and intakes are lowest when available under predictable schedules (Lagorio & Winger, 2014; Zamarripa et al., 2023). Taken together, prior research suggests that finding ways to reduce the unpredictability associated with drug access is a worthy endeavor for policy and treatment of SUDs, and this can be achieved through agonist-medication approaches or safer drug supplies. The outcomes reviewed also suggest that finding ways to reduce the unpredictability of nondrug commodities or making nondrug alternatives in the environment much easier to obtain can reduce the impact of unpredictable access to drugs on drug taking through incentive-based programs like contingency management or a community reinforcement approach. In the next few paragraphs, we will describe each of these policy or treatment implications.

Agonist medications are available for opioid use disorders (i.e., methadone, buprenorphine) and nicotine dependence (i.e., nicotine replacement therapies). These medications are intended to alleviate withdrawal and craving for misused drugs via similar pharmacological mechanisms as the misused drug. Furthermore, these medications typically are relatively long-acting drugs so that maintenance on the medication, at least for opioids, can be achieved with once/daily dosing or sustained release injectables. Importantly, agonist medications are effective treatments for opioid use disorder; such medications improve treatment retention and reduce mortality, transmission of infectious disease, and drug-related crime (e.g., Kakko et al., 2003; Mattick et al., 2009; NIDA, 2021a). While the intention of agonist medications is to reduce withdrawal and craving, this approach also could be effective because it makes access to a pharmacologically similar substance predictable. Based on the research reviewed above, making agonist medications available under small time and effort requirements and short delays to delivery would make them more effective treatments than if their availability requires large time and effort requirements or long delays to obtaining the medication. Furthermore, it is important that the dose of the agonist medication is sufficiently high to compete with unpredictable illicit-drug sources, and several recent clinical papers have confirmed that higher doses of both methadone and buprenorphine result in better treatment outcomes than moderate or low doses (e.g., Axeen et al., 2024; Fareed et al., 2009, 2012; Strain et al., 1993, 1999). Once an agonist medication is available in a predictable way, at a sufficiently large dose, one can dedicate more time to nondrug-related activities. Another important aspect of agonist medications is that these medications are safer than illicit-drug supplies as overdose rates and emergency room visits are much lower with agonist medications than with illicit drugs (Hedegaard et al., 2018; NIDA, 2021b). It is possible that an agonist-medication approach would be similarly effective for other drug classes, for example, these medications have been proposed for stimulant use disorders (e.g., Negus & Henningfield, 2015; Stoops & Rush, 2013).

Like agonist medications, safer supply programs also would make access to misused drugs more predictable. While this approach is admittedly controversial, the “War on Drugs” and strategies aimed at eliminating drugs from the illicit market do not reduce drug taking. In fact, when certain drugs become more difficult or impossible to obtain, dealers often resort to synthetic analogs to evade drug laws (e.g., Tamama, 2021; Tyndall, 2020). This results in an illicit drug supply that is adulterated with synthetic substances with unknown health risks, and in the case of fentanyl, has resulted in a deadlier drug-overdose crisis compared with the prescription medication or heroin overdose crises that preceded the widespread availability of fentanyl and other synthetic opioids (e.g., see Ciccarone, 2019 for a discussion of the three waves).

Creating a safer drug supply through the prescription of drugs that are considered illicit or through legal regulation of such drugs would make access to substances more predictable and safer. Like agonist medications, a safer supply reduces crime and overdose rates and improves health outcomes, and drug use patterns (e.g., Bernstein et al., 2020; Fairgrieve et al., 2020; Fleming et al., 2020; Kolla et al., 2024). For example, users of a safer supply report reducing their use of drugs from the unregulated, illicit market and report that they are able to manage withdrawal, pain, and cravings with their safer supply if users are able to access sufficiently high doses of certain substances (Kolla et al., 2024). Furthermore, participants in Kolla and colleagues’ study reported an appreciation for the safer supply services, and perhaps most importantly, felt that the treatment was lifesaving, providers were compassionate, and accessing the safer supply program was non-stigmatizing. Participants also reported improved function and quality of life, and they reported that they reduced their use of illicit methamphetamine or fentanyl when they had access to safer supplies of stimulant or opioid medications. Finally, participants reported that accessing a safer supply meant that they could avoid the “hustle”; participants reported not having to steal to get drugs, and that they no longer had to hustle for money all the time which resulted in more time to do things that they enjoyed.

Finally, outcomes reviewed above have implications for contingency management. Contingency management is a therapy that can be leveraged to target many behaviors, including the misuse of illicit substances, and is the most effective psychosocial therapy for SUDs (Dutra et al., 2008; Prendergast et al., 2006). In contingency management, nondrug reinforcers, often in the form of vouchers exchangeable for commodities or goods and services, are delivered to participants contingent on objective and verifiable drug abstinence. Based on the literature reviewed above, contingency management will most effectively compete with unpredictable illicit-drug access when the vouchers are available under relatively small time and effort requirements. Furthermore, contingency management may be able to leverage what is known about unpredictable delivery of nondrug reinforcers and their ability to compete with drug reinforcers. If unpredictable amounts garner more behavior, unpredictable voucher amounts also should have this effect on behavior. Indeed, prize-based contingency management varies the probability and magnitude of vouchers exchangeable for goods or prizes contingent on objective and verifiable drug abstinence (e.g., Petry & Alessi, 2010; Petry & Martin, 2002; Petry et al., 2000, 2005a, 2005b). Prize-based contingency management is as effective as standard contingency management, even though the expected amount of reinforcement for perfect performance is lower with prizes compared with standard contingency management (Petry et al., 2005a). In addition to providing an effective treatment for SUDs, prize-based contingency management is more cost effective than standard contingency management (Olmstead et al., 2007). Furthermore, contingency management is often used in combination with agonist medications and could certainly be incorporated into a safer supply program.

As indicated at the beginning of this review, several environmental and genetic factors contribute to drug use, misuse, and the development of SUDs. Unpredictable access to illicit drugs in real-world situations may be one of the environmental factors that contributes to worsened drug-related outcomes compared with more predictable access. However, as noted above, the research reviewed also demonstrated that such detrimental effects on drug-related outcomes often were dependent on the environmental conditions in which drugs are available. More research is needed to further elucidate which conditions and behavioral mechanisms are responsible for outcomes with unpredictable drug access, as well as which conditions reduce the detrimental effects of unpredictable drug access that may be valuable for policy and treatment.
